# Astaxanthin Protects Dendritic Cells from Lipopolysaccharide-Induced Immune Dysfunction

**DOI:** 10.3390/md19060346

**Published:** 2021-06-17

**Authors:** Yinyan Yin, Nuo Xu, Yi Shi, Bangyue Zhou, Dongrui Sun, Bixia Ma, Zhengzhong Xu, Jin Yang, Chunmei Li

**Affiliations:** 1 College of Medicine, Yangzhou University, Yangzhou 225009, China; kf56xunuo58@126.com (N.X.); sy15365888238@163.com (Y.S.); zby18252737828@163.com (B.Z.); sun1316561047@163.com (D.S.); 2 Jiangsu Key Laboratory of Experimental and Translational Non-Coding RNA Research, Yangzhou University, Yangzhou 225009, China; 3 Clinical Medical College, Yangzhou University, Yangzhou 225001, China; 4 College of Food Science and Engineering, Yangzhou University, Yangzhou 225009, China; mabixia@vazyme.com; 5 Jiangsu Key Laboratory of Zoonosis, Yangzhou University, Yangzhou 225009, China; zzxu@yzu.edu.cn

**Keywords:** astaxanthin, dendritic cells, sepsis, immune dysfunction, lipopolysaccharide

## Abstract

Astaxanthin, originating from seafood, is a naturally occurring red carotenoid pigment. Previous studies have focused on its antioxidant properties; however, whether astaxanthin possesses a desired anti-inflammatory characteristic to regulate the dendritic cells (DCs) for sepsis therapy remains unknown. Here, we explored the effects of astaxanthin on the immune functions of murine DCs. Our results showed that astaxanthin reduced the expressions of LPS-induced inflammatory cytokines (TNF-α, IL-6, and IL-10) and phenotypic markers (MHCII, CD40, CD80, and CD86) by DCs. Moreover, astaxanthin promoted the endocytosis levels in LPS-treated DCs, and hindered the LPS-induced migration of DCs via downregulating CCR7 expression, and then abrogated allogeneic T cell proliferation. Furthermore, we found that astaxanthin inhibited the immune dysfunction of DCs induced by LPS via the activation of the HO-1/Nrf2 axis. Finally, astaxanthin with oral administration remarkably enhanced the survival rate of LPS-challenged mice. These data showed a new approach of astaxanthin for potential sepsis treatment through avoiding the immune dysfunction of DCs.

## 1. Introduction

The immune system, as a tight and dynamic regulatory network, maintains an immune homeostasis, which keeps a balance between the response to heterogenic antigens and tolerance to self-antigens [1]. However, in some diseases, such as sepsis, rheumatoid arthritis (RA), multiple sclerosis (MS), systemic lupus erythematosus (SLE), and inflammatory bowel disease (IBD), this immune homeostasis is broken [2]. Sepsis is a highly heterogeneous clinical syndrome that mainly results from the dysregulated inflammatory response to infection, which continues to cause considerable morbidity and accounts for 5.3 million deaths per year in high income countries [3]. Recently, the incidence of sepsis is progressively increased and sepsis-related mortality cases remain at a high level in China [4]. The host immune response induced by sepsis is a complex and dynamic process. After infection, the conserved motifs of pathogens, termed the pathogen-associated molecular patterns (PAMPs), such as lipopolysaccharide (LPS, cell wall component of gram-negative bacteria) or lipoteichoic acid (cell wall component of gram-positive bacteria), are recognized by the pattern recognition receptors (PRRs) expressed by immune cells, and an overwhelming innate immune response is triggered in septic patients [5,6]. Under physiological conditions, the immune activation contributes to eliminating pathogens and clearing infected cells. However, when driven by sepsis, the immune homeostasis appears imbalanced and initiates a life-threatening “cytokine storm”. Currently, no drugs have been approved specifically for the treatment of sepsis, and clinical trials of potential therapies have failed to reduce mortality; therefore, new approaches are needed. Immune-modulatory intervention is the main potential therapeutic strategy against sepsis [7]. For instance, single cytokine, or a combination of multiple cytokines, including G-CSF, GM-CSF, IFN-γ, IL-3, IL-7, and IL-15, were introduced into sepsis therapy, according to a disease-specific progression and patient immune responses [8]. Some immunosuppressive agents, such as bursopentin [9], curcumin [10], and oleuropein [11], provided protection against inflammatory injury in the LPS-induced sepsis models.

Dendritic cells (DCs), as the most important potent antigen-presenting cells, link the innate and adaptive immune response. The maturation/activation of DCs is followed by the transformations of phenotype and function, improving their migration ability to draining the lymph node, resulting in the activation of downstream T lymphocyte cells [12]. In fact, DCs reside in all the tissues of the host mainly in an antigen-capturing state and maintain immune tolerance by migrating to the lymph nodes for presenting self-antigens to lymphocytes in a tolerogenic manner [13]. Therefore, DCs also balance the immune homeostasis in the host, and guide the skewing of the downstream immune response [14]. Notably, the abnormalities of DC homeostasis are implicated in sepsis. The differentiation level of monocytes into DCs is improved during sepsis [15]. The expression levels of surface molecules related to the DC function are changed [16]. Considering the critical role of DCs in the immune regulation in sepsis, the modification of the DC system is becoming an increasingly important target for sepsis therapy [15]. Modificatory DCs by adenovirus/IL-10 transduction maintained an immature state with low expressions of IL-12, CD86, and MHCII, and the survival rate of septic mice remarkably increased [17,18]. Bursopentin inhibited the LPS-induced phenotypic and the functional maturation of DCs [9,19]. These studies indicated that compartmental modification of DC function can alter the sepsis-induced immune response.

Astaxanthin, 3,3′-dihydroxy-β,β′-carotene-4,4′-dione, is a naturally occurring red carotenoid pigment classified as a xanthophyll, found in microalgae and seafood such as salmon, trout, and shrimp [20,21]. The lipid-soluble carotenoid, with a polar–nonpolar–polar structure, is able to help astaxanthin easily pass through and fix into the double layers of the cell membrane. Moreover, free radicals inside and outside of the cell membrane can be scavenged by the polar structure of astaxanthin, and radicals located in the cell membrane can be captured by its polyene chain [22]. Therefore, astaxanthin has a strong antioxidant property, and is regarded as a potential candidate agent against many diseases [23,24,25,26]. Recent studies have shown that astaxanthin had a variety of pharmacological effects against inflammatory injury [27,28,29]. Astaxanthin provided a neuroprotection against diabetes-induced sickness behavior through inhibiting inflammation [30]. Astaxanthin also can attenuate monosodium urate crystal-induced arthritis by suppressing the level of pro-inflammatory cytokines [31]. Moreover, astaxanthin was shown to suppress LPS-induced inflammatory factors increase, MAPK phosphorylation, and NF-kB activation in vivo [32]. These studies demonstrated to us that astaxanthin have a great potential as a therapeutic agent of sepsis by an anti-inflammatory strategy.

In this study, we attempted to characterize the effects of astaxanthin on the immune activation and functional properties of the LPS-induced DCs for potential sepsis therapy. Our data suggested that astaxanthin protected DCs from LPS-induced immune dysfunction, which might be a simple, inexpensive, and highly effective anti-inflammatory strategy via regulating DC activity in sepsis.

## 2. Results

### 2.1. Astaxanthin Inhibited LPS-Induced Cytokine Production by DCs

**Figure 1:**
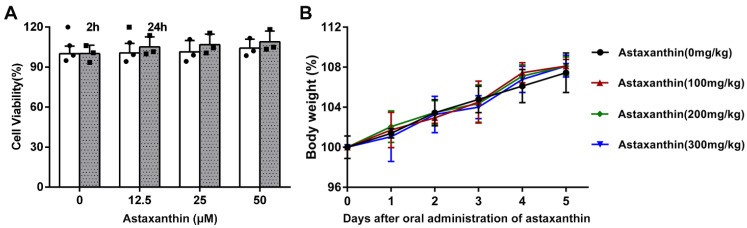
Biosafety evaluation of astaxanthin in vitro and in vivo. (**A**) The cytotoxicity of astaxanthin with different doses was performed in the DCs by using the CCK-8 assay. (**B**) Astaxanthin with different concentrations was given orally for five days every 24 h; the data represent the change of body weight in each group (*n* = 10/group). The data shown are the means ± s.d. of three replicates and are representative of three independent experiments. Statistical significance is assessed by unpaired Student’s two-sided *t*-test to compare astaxanthin (0 μM).

**Figure 2:**
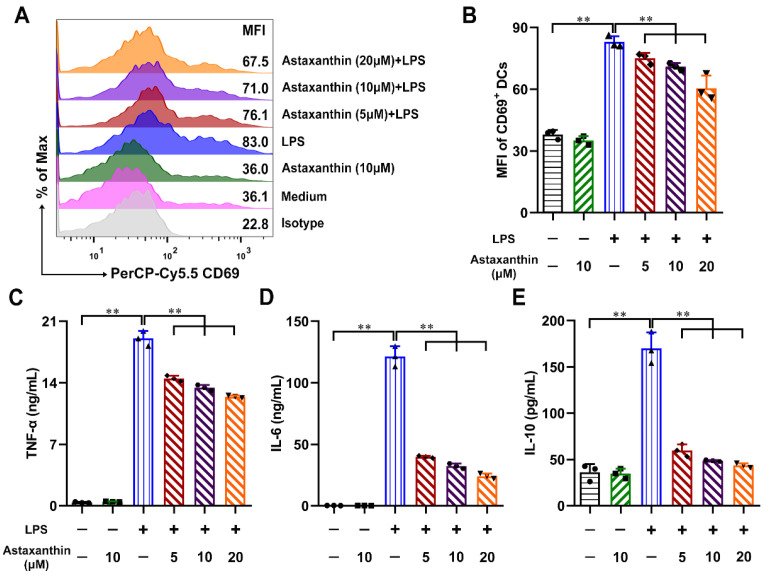
Astaxanthin suppressed the secretion of cytokines from LPS-stimulated DCs. DCs were incubated with the astaxanthin or plus 100 ng/mL LPS for 24 h. (**A**,**B**) The expression of activation marker CD69 on DCs was analyzed by FCM. (**C**–**E**) Supernatants were collected and TNF-α, IL-6, and IL-10 were detected by ELISA. The data shown are the means ± s.d. of three replicates and are representative of three independent experiments. Statistical significance is assessed by one-way ANOVA analysis to compare the results between different groups. ** *p* < 0.01.

Firstly, the biosafety of astaxanthin was evaluated in the murine DCs. The cells were treated with astaxanthin and the cell viability was analyzed by the CCK-8 assay. The results revealed that the cellular viability was not changed until 24 h after treatment with astaxanthin up to 50 μM (Figure 1A). Next, we examined the expression of CD69, which is a critical activation marker of DCs. After exposure to LPS (100 ng/mL) for 24 h, the expression of CD69 was upregulated, whereas they were significantly inhibited with treatment of astaxanthin (Figure 2A,B). In addition, we tested whether astaxanthin affected the production of cytokines in LPS-induced DCs. Significantly, pro-inflammatory cytokines (TNF-α and IL-6) were downregulated by astaxanthin in a dose-dependent manner (Figure 2C,D). Surprisingly, the secretion of IL-10 was not increased (Figure 2E), implying that the suppressive effect of astaxanthin probably was not mediated through anti-inflammatory cytokine. These results indicated that astaxanthin attenuated the cytokines secreted by LPS-induced DCs.

### 2.2. Astaxanthin Reversed the Morphological Changes in LPS-Activated DCs

**Figure 3:**
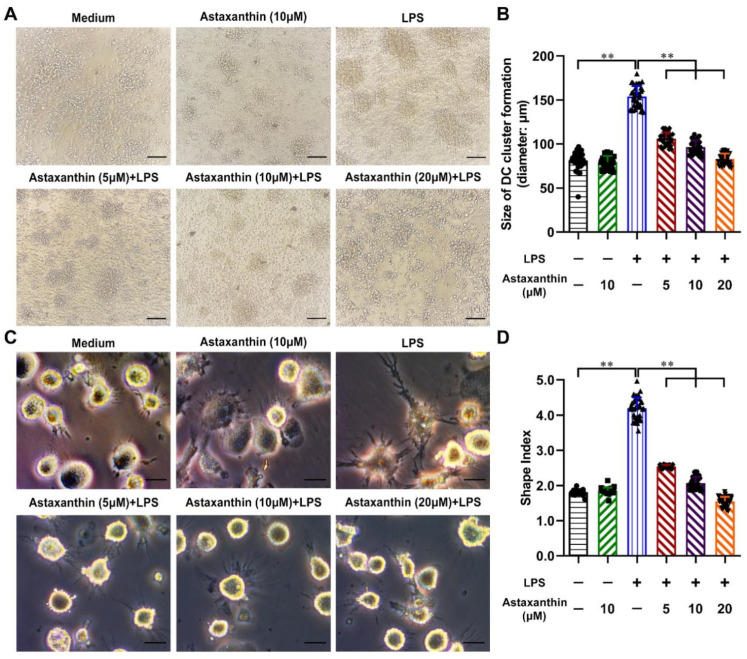
Astaxanthin decreased the morphological changes of LPS-stimulated DCs. After stimulation for 24 h with astaxanthin or plus 100 ng/mL LPS, DC aggregation (**A**) and dendrites (**C**) were observed by microscopy. (**B**,**D**) Statistical results on the size of DC cluster formation and the cellular shape indices in each group. Data shown are the means ± s.d. of 40 clusters or DCs randomly selected from 3 separate experiments. Statistical significance is assessed by one-way ANOVA analysis to compare the results between different groups. ** *p* < 0.01. Bars: (**A**) 100 μm; (**C**) 20 μm.

Mature DCs were easily aggregated to form larger clusters and longer extensions [33]. Upon LPS stimulation alone, the size of clusters and the extension morphologies of DCs were increased, compared with the untreated and the astaxanthin-alone group. However, these processes were impaired by astaxanthin (Figure 3A,C). Meanwhile, the size of clusters and the cell shape index (major axis/minor axis) of each group were measured. As shown in Figure 3B,D, these two indexes were markedly increased after LPS stimulation. Treatment of astaxanthin significantly suppressed the increase of two indexes in LPS-induced DCs. These results indicated that astaxanthin attenuated the morphological changes of LPS-activated DCs.

### 2.3. Astaxanthin Impaired the Phenotypic Maturation of LPS-Induced DCs

**Figure 4:**
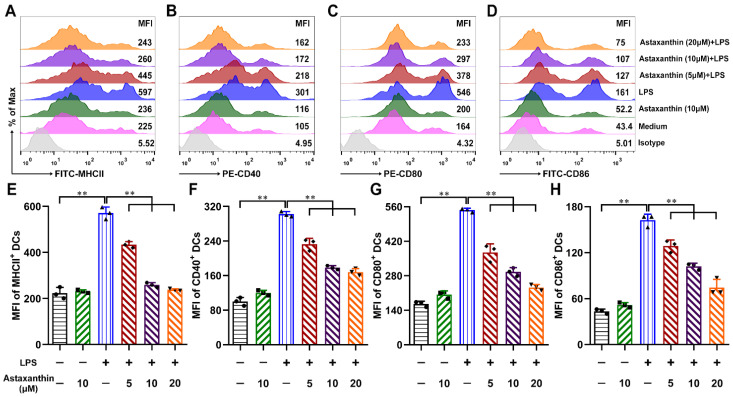
Astaxanthin suppressed the expression of phenotypic markers by LPS-stimulated DCs in vitro. After stimulation for 24 h with astaxanthin or plus 100 ng/mL LPS, the expressions of phenotypic markers on DCs, including MHCII (**A**,**E**), CD40 (**B**,**F**), CD80 (**C**,**G**), and CD86 (**D**,**H**), were analyzed by FCM. Data shown are the means ± s.d. of three replicates and are representative of three independent experiments. Statistical significance is assessed by one-way ANOVA analysis to compare the results between different groups. ** *p* < 0.01.

Maturation is the key step in the DC-mediated regulation of immune responses. To investigate whether astaxanthin modulated the DC maturation, the expression levels of MHCII and costimulatory molecules in DCs were analyzed by FCM. With LPS treatment alone, the expressions of MHCII, CD40, CD80, and CD86 were markedly upregulated, whereas they were down-regulated remarkably with the treatment of astaxanthin (Figure 4). These data suggested that astaxanthin diminished LPS-activated DC phenotypic maturation and compromised the immunostimulation of the activated DCs.

### 2.4. Astaxanthin Increased the Endocytosis Capability of LPS-Induced DCs

**Figure 5:**
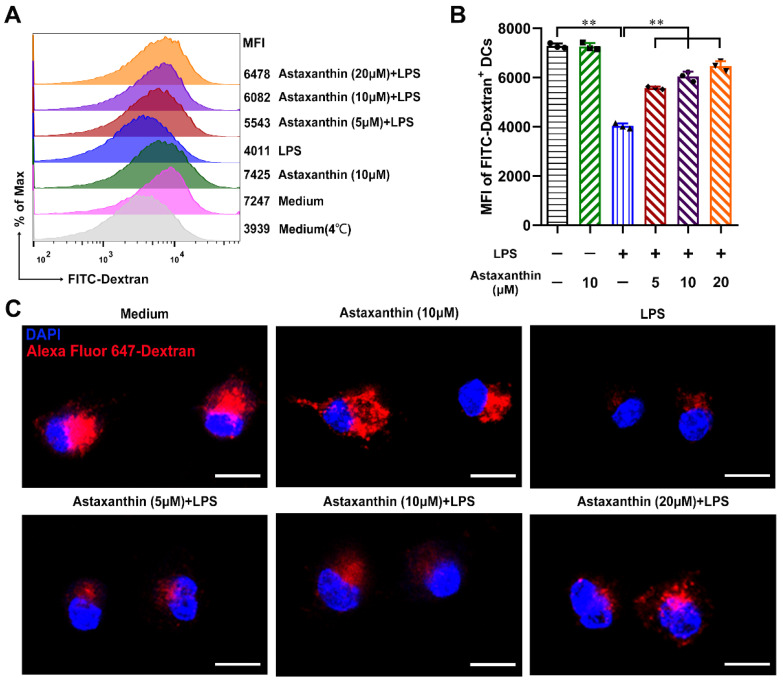
Astaxanthin enhanced the endocytosis ability of DCs after LPS treatment in vitro. After stimulation for 24 h with astaxanthin or plus 100 ng/mL LPS, the treated DCs were incubated with 1 mg/mL FITC-Dextran (**A**,**B**) or Alexa Fluor 647-Dextran (**C**) at 37 °C for 30 min. After incubation, the cells were washed three times with cold PBS and analyzed by FCM (**A**) or were observed by using confocal laser scanning microscopy (CLSM). Parallel experiments were performed at 4 °C to determine the nonspecific binding. The data shown are the means ± s.d. of three replicates and are representative of three independent experiments. (**C**) Dextran (Alexa Fluor 647; red) and Nuclei (4′,6-diamidino-2-phenylindole (DAPI); blue). The results are from one representative experiment of three performed. Bars: 10 μm. Statistical significance is assessed by one-way ANOVA analysis to compare the results between different groups. ** *p* < 0.01.

In response to inflammatory stimuli, DCs trigger the process of maturation; down-regulation of endocytosis is a hallmark of maturation [34]. To investigate whether astaxanthin modulated the endocytosis of DCs, the fluorescent marker dextran was used. As shown in Figure 5A,B, LPS alone significantly decreased the endocytosis capability of DCs compared to the untreated control, while astaxanthin enhanced the uptake of dextran in LPS-induced DCs. Moreover, confocal laser scanning microscopy (CLSM) images displayed the amount of Alexa Fluor 647-dextran existing in the body of LPS-induced DCs and was enhanced after the treatment of astaxanthin (Figure 5C). These results suggested that astaxanthin significantly increased the endocytosis capability of LPS-induced DCs.

### 2.5. Astaxanthin Inhibited the Migration Capability of LPS-Induced DCs

**Figure 6:**
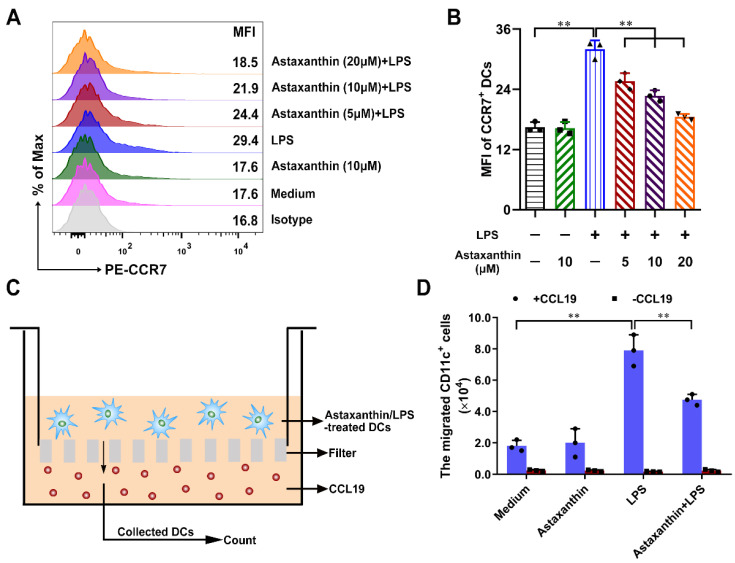
Astaxanthin decreased the LPS-induced CCR7 expression and DC’s migration ability in vitro. DCs were incubated by astaxanthin or plus 100 ng/mL LPS for 24 h. (**A**,**B**) FCM analysis of CCR7 expression. Data shown are the means ± s.d. of three replicates and are representative of three independent experiments. (**C**,**D**) DCs from astaxanthin (10 μM) alone, LPS (100 ng/mL) alone, astaxanthin (10 μM) plus LPS (100 ng/mL) groups were seeded into the upper wells of a 24-well transwell chamber, and CCL19 (200 ng/mL) was included in lower chamber. After 4 h, the number of DCs that were transferred from the upper to the lower wells was counted by FCM. The spontaneous migration of cells (absence of CCL19) was also shown. Data shown are the means ± s.d. of three replicates and are representative of three independent experiments. Statistical significance is assessed by one-way ANOVA analysis to compare the results between different groups. ** *p* < 0.01.

DCs that are stimulated with inflammatory mediators can mature and migrate from nonlymphoid regions to lymphoid organs for initiating T cell-mediated immune responses. This migratory step is closely related to the CCR7 expression of DCs [35]. To investigate whether astaxanthin modulated the DC migration, the expression levels of CCR7 in DCs were analyzed by FCM. With LPS treatment alone, CCR7 expression was significantly increased, whereas they remarkably declined after the treatment of astaxanthin (Figure 6A,B). Moreover, chemotaxis assay in transwell chambers was used to examine the DC migration on the basis of attraction of mature DCs for CCL19 or CCL21. The migration of LPS-induced DCs was remarkably inhibited after the treatment of astaxanthin in response to CCL19 (Figure 6C,D). These results suggested that astaxanthin significantly inhibited the migration capability of LPS-induced DCs.

### 2.6. Astaxanthin Impaired the Allostimulatory Capacity of LPS-Induced DCs

**Figure 7:**
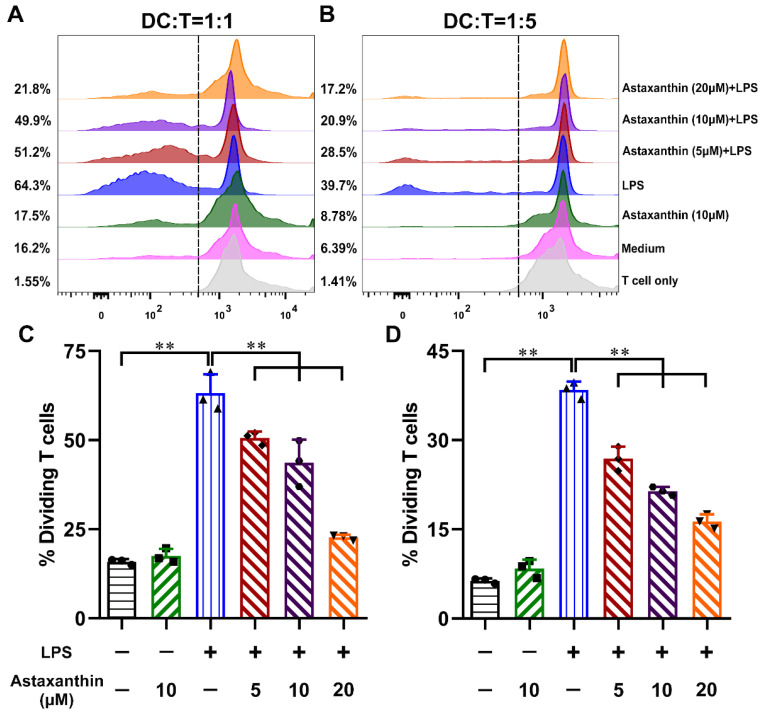
Astaxanthin decreased LPS-induced DCs to increase the proliferation of allogeneic T cells. After incubation with astaxanthin or plus 100 ng/mL LPS for 24 h, the collected DCs were used in two graded cell numbers (DC/T-cell ratios: 1:1 (**A**,**C**) and 1:5 (**B**,**D**)) to stimulate CFSE-labeled naive CD4^+^ allogeneic T cells (5 × 10^5^ responder cells per well). After 5 days, proliferation was detected by FCM. Data shown are the means ± s.d. of three replicates and are representative of three independent experiments. Statistical significance is assessed by one-way ANOVA analysis to compare the results between different groups. ** *p* < 0.01.

Mature DCs are potent stimulators of allogeneic T cell proliferation in the mixed lymphocyte reaction (MLR) [36]. To determine the effects of astaxanthin on the ability of LPS-induced DCs to stimulate the MLR, DCs were collected and incubated with allogeneic CD4^+^ T cells. As shown in Figure 7, LPS-induced DCs stimulated proliferative responses more effectively than untreated DCs, while astaxanthin-treated DCs impaired proliferative responses derived from the LPS stimulation at all ratios of DC: T cell tests. These results suggested that astaxanthin strongly impaired the allostimulatory capacity of LPS-induced DCs.

### 2.7. Astaxanthin Protected the LPS-Induced Immune Dysfunction of DCs via Activation of HO-1/Nrf2 Axis

**Figure 8:**
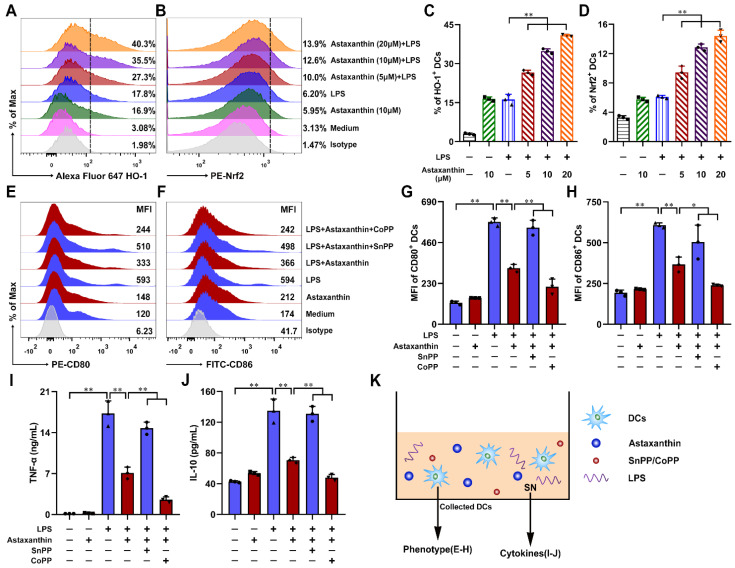
Astaxanthin enhanced the expression of HO-1 and Nrf2 protein in the LPS-induced DCs. (**A**–**D**) DCs were incubated by astaxanthin or plus 100 ng/mL LPS for 24 h. FCM analysis of HO-1 and Nrf2 expression. Data shown are the means ± s.d. of three replicates and are representative of three independent experiments. (**K**) Experimental setting to study DC maturation, DCs were treated with 10 μM astaxanthin or plus 100 ng/mL LPS in the presence or absence of SnPP (25 μM) or CoPP (50 μM) for 24 h. (**E**–**H**) The expressions of CD80 (**E**,**G**) and CD86 (**F**,**H**) were detected by FCM. (**I**,**J**) TNF-α and IL-10 released from supernatants were detected by ELISA. The data shown are the means ± s.d. of three replicates and are representative of three independent experiments. Statistical significance is assessed by one-way ANOVA analysis to compare the results between different groups. * *p* < 0.05; ** *p* < 0.01. SN: supernatant.

To investigate whether astaxanthin modulated the DC maturation by the HO-1/Nrf2 pathway, the expression levels of HO-1 and Nrf2 on DCs were analyzed by FCM. As shown in Figure 8A–D, treatment of LPS-induced DCs with astaxanthin, HO-1, and Nrf2 were significantly upregulated, compared with the LPS-only group. Next, to study whether HO-1 played an important role in the suppression of DC maturation, the cytokine release (TNF-α and IL-10) (Figure 8I,J) and phenotypic markers (CD80 and CD86) (Figure 8E–H) were detected. The results showed that the effects of astaxanthin in the LPS-induced DCs were diminished when DCs were pretreated with SnPP (a HO-1 inhibitor) (Figure 8E–J). However, CoPP (a HO-1 inducer) aggravated the inhibitory effect of astaxanthin in the LPS-induced DCs (Figure 8E–J). Therefore, the Nrf2/HO-1 pathway played an important role in the inhibition of LPS-induced DCs maturation by astaxanthin.

### 2.8. Astaxanthin Protected LPS-Induced Sepsis in Mice

**Figure 9:**
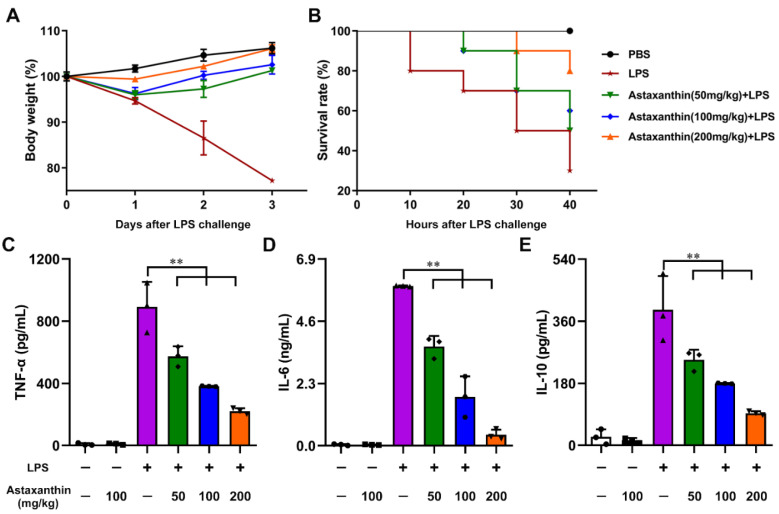
Astaxanthin recovered the changes in body weight and decreased the cytokines secretion in LPS-challenged mice. (**A**,**B**) The data represent the body weight changes and survival rates of each group (*n* = 10/group). (**C**–**E**) The level of cytokines in plasma was measured by ELISA. Data shown are the means ± s.d. of three replicates and are representative of three independent experiments. Statistical significance is assessed by one-way ANOVA analysis to compare the results between different groups. ** *p* < 0.01.

The overwhelming production of pro-inflammatory cytokines and mediators results in tissue damage or lethality. To determine the effects of astaxanthin on the LPS-induced septic lethal rate and production of cytokines in LPS-challenged mice, firstly, the biosafety of astaxanthin was evaluated in mice. As shown in Figure 1B, the body weight of mice was not changed in the astaxanthin group compared with the control group, even if the dose used was up to 300 mg/kg. Next, the changes in body weight and survival rates were monitored after LPS injection for 3 days or 40 h, respectively. As shown in Figure 9A, LPS administration markedly increased the loss of body weight in mice. However, the astaxanthin recovered the change of body weight in the LPS-challenged mice. Moreover, the astaxanthin decreased the mortality of the LPS-treated mice (Figure 9B). Next, the levels of cytokines in mice serum were detected by ELISA. The results showed that administration of astaxanthin significantly decreased the production of TNF-α, IL-6, and IL-10 (Figure 9C–E). Taken together, these data demonstrated that astaxanthin effectively protected LPS-induced sepsis in mice.

## 3. Discussion

**Figure 10:**
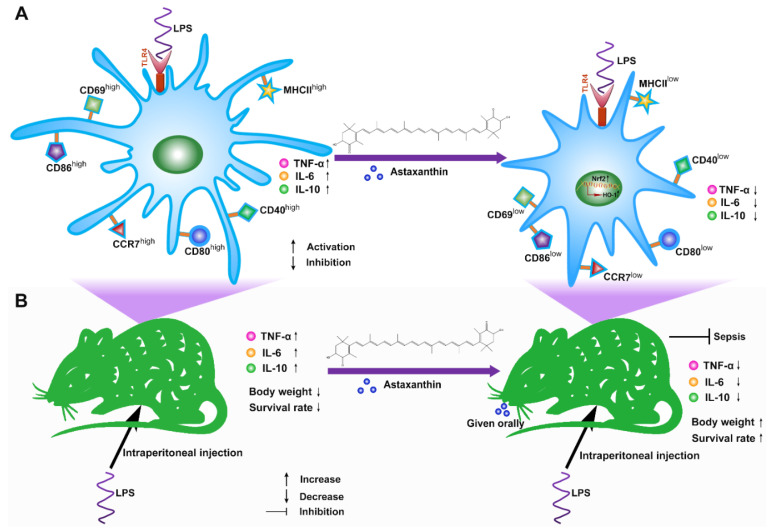
Schematic of the proposed mechanism for astaxanthin, rescuing the LPS-induced immune dysfunction of DCs and protecting LPS-induced sepsis in mice. (**A**) Astaxanthin firstly activated the Nrf2 signaling pathway, and then significantly upregulated HO-1 expression, which suppressed the immune functions of LPS-induced DCs, including activation markers (CD69), the cytokines release (TNF-α, IL-6, and IL-10), phenotypic marker (MHCII, CD40, CD80, and CD86) and migration marker (CCR7). (**B**) Astaxanthin decreased the production of TNF-α, IL-6, and IL-10 in serum, recovered the change in body weight and decreased the mortality of the LPS-treated mice.

Here, we explored the immunosuppressive properties of astaxanthin on the activation and maturation of DCs for the first time. Our data indicated that astaxanthin reduced the expression of activation markers (CD69), LPS-induced pro-inflammatory (TNF-α and IL-6), and anti-inflammatory (IL-10) cytokines by DCs; reversed the morphological changes of LPS-activated DCs; decreased the LPS-induced expression of phenotypic markers by DCs, including MHCII, CD40, CD80, and CD86; promoted the endocytosis levels in LPS-treated DCs; and hindered the LPS-induced migration of DCs via downregulating CCR7 expression. Furthermore, astaxanthin abrogated allogeneic T cell proliferation by LPS-induced DCs. Finally, astaxanthin enhanced the survival rate of LPS-challenged mice and inhibited the production of inflammatory cytokines in serum, suggesting that astaxanthin can strongly protect LPS-induced sepsis (Figure 10).These results powerfully implied that astaxanthin may have a potential application in the treatment of sepsis.

Toll-like receptor (TLR) 4 signaling, leading to secretion of inflammatory productions, has been considered as a critical pathway in sepsis pathophysiology. LPS from gram-negative bacteria interacted with TLR4 to cause phagocytic cells to robustly generate a variety of proinflammatory cytokines [37]. CD69, as a type II C-type lectin, is known as a very early activation marker, which is first upregulated upon primary activation [38,39]. In our study, we found that astaxanthin reduced the activation level of LPS-treated DCs by downregulating CD69 expression, suggesting that the immunosuppressive ability of astaxanthin was involved in the early inflammatory response. After DC activation, a mass of inflammatory cytokines was released. TNF-α, as a rapid proinflammatory cytokine, can strongly accelerate DC maturation [40]. Furthermore, TNF-α also can regulate other inflammatory cytokines, especially for IL-6 [41], implying that astaxanthin might suppress the secretion of TNF-α, and then result in the down-expression of IL-6 in DCs. At the late stage of sepsis, the anti-inflammatory state may appear, showing a high expression of IL-10, which may result in a further impaired immune response with an increased risk of nosocomial infections [42]. Therefore, we evaluated the effects of astaxanthin treatment in LPS-induced IL-10 expression, and found that IL-10 was also decreased, and thereby, astaxanthin plays a remarkable inhibition role on both pro- and anti-inflammatory stages.

DCs possess two major states, including immature DCs (iDCs) and mature DCs (mDCs). The iDCs have a strong antigen capture ability with lower expression of phenotypic markers. After antigen uptake, iDCs were transformed into mDCs, which have a strong ability to stimulate the proliferation and differentiation of T cells by upregulating the surface levels of MHCII and costimulatory molecules. Moreover, DCs can easily mature into inflammatory DCs, thereby sustaining a continuous activation of the adaptive immune response at inflammation sites [43]. However, iDCs were able to induce immune tolerance, and have therefore been introduced as a therapy for systemic lupus erythematosus (SLE) [44,45]. In our data, astaxanthin can effectively inhibit LPS-induced phenotypic markers of DCs, including MHCII, CD40, CD80, and CD86, suggesting that astaxanthin was able to prevent the transformation from iDCs into mDCs. In addition, LPS-induced DCs with astaxanthin treatment possessed a strong antigen capture ability, indicating that the DCs remain in an immature state. Furthermore, once DCs mature, the chemokine receptor CCR7 displays a high-upregulation, which will guide the DCs to migrate toward a draining lymph node, a T cell-rich area with a high expression of CCL19 and CCL21 (CCR7 ligands), for an expanded immune response [46]. Our data suggested that astaxanthin could probably block the connection between DCs and draining lymph nodes via down-regulating CCR7 expression, and lead to limit extensive immune responses. Even if contact happened, LPS-induced DCs with astaxanthin treatment were hardly promoted to a proliferation of allogeneic T cells in our allogeneic mixed lymphocyte reaction assay, which might be associated with the down-regulation of MHCII, costimulatory molecules, and cytokines.

Inflammation is the most common feature of many chronic diseases and complications. Previous studies have revealed that the transcription nuclear factor erythroid 2-related factor 2 (Nrf2) contributes to the anti-inflammatory process by orchestrating the recruitment of inflammatory cells and regulating gene expression through the antioxidant response element (ARE) [47]. Heme oxygenase-1 (HO-1) is the inducible isoform and rate-limiting enzyme that catalyzes the degradation of heme into carbon monoxide (CO) and free iron, and biliverdin to bilirubin [48]. Several studies have demonstrated that HO-1 and its metabolites have significant anti-inflammatory effects mediated by Nrf2 [49]. It has been reported that activation of Nrf2 prevents LPS-induced transcriptional upregulation of pro-inflammatory cytokines, including IL-6 and IL-1β [50]. Here, we have demonstrated that astaxanthin inhibited the maturation of LPS-induced DCs via the activation of the HO-1/Nrf2 axis. Interestingly, astaxanthin is a potential antioxidant, and the HO-1/Nrf2 axis is also a key known antioxidative pathway; whether astaxanthin utilizes its antioxidant property to activate the HO-1/Nrf2 pathway and then to initiate an anti-inflammatory response needs to be further investigated.

LPS and other PAMPs are related in the pathogenesis of sepsis and the activation of immune responses, resulting in tissue pathological injury and multiple organ failure [51]. Management of excessive inflammatory response is a key strategy for sepsis treatment [52]. In the present study, we performed a series of experiments to determine the anti-inflammatory activities of astaxanthin using LPS-challenged mice. Our results showed that administration of astaxanthin promoted the survival rate of LPS-challenged mice. Additionally, administration of astaxanthin reduced the levels of inflammatory cytokines in serum, including TNF-α, IL-6, and IL-10, which was in line with the result of DCs in vitro. These results implied that DC-targeted anti-inflammatory strategies have great potential in the treatment of sepsis.

## 4. Materials and Methods

### 4.1. Ethics Statement

The Jiangsu Administrative Committee for Laboratory Animals approved all of the animal studies according to the guidelines of Jiangsu Laboratory Animal Welfare and Ethical of Jiangsu Administrative Committee of Laboratory Animals (Permission number: SYXKSU-2007-0005).

### 4.2. Reagents

Astaxanthin (mol wt 596.84), LPS derived from *Escherichia coli* 026: B6, FITC-Dextran (mol wt 40,000) and Cobalt protoporphyrin (CoPP, a HO-1 inducer) were from Sigma-Aldrich. Alexa Fluor 647-Dextran (mol wt 10,000) was from Thermo Fisher. Carboxyfluorescein succinimidylester (CFSE) and RPMI 1640 medium were from Invitrogen. Fetal bovine serum (FBS) was from Hyclone. Recombinant CCL19, GM-CSF, and IL-4 were from Peprotech. CCK-8 kit was from Beyotime. CD4^+^ T cell isolation kit was from Miltenyi Biotech. Fluorescent-labeled anti-mouse mAbs, PerCP-Cy5.5 CD69, FITC-MHCII, PE-CD40, PE-CD80, FITC-CD86, PE-CCR7 or respective isotype controls, were from BD PharMingen. Alexa Fluor 647 HO-1 or respective isotype was from Abcam. PE-Nrf2 or respective isotype was from Cell Signaling Technology. Tin protoporphyrin IX (SnPP, a HO-1 inhibitor) was from MedChemExpress.

### 4.3. Generation of DCs

Male C57BL/6 mice, 4–6 weeks old, were from the Animal Research Center of Yangzhou University (Jiangsu, China). The mice were housed under specific pathogen-free conditions for at least 1 week before use. DCs were isolated and cultured as our improved method [53]. Briefly, bone marrow cells were extracted from the tibias and femurs of mice, and then cultured in complete medium (RPMI 1640 supplemented with 10% FBS, 1% streptomycin and penicillin, 10 ng/mL GM-CSF and 10 ng/mL IL-4). After 60 h of culture, medium was gently discarded and fresh medium was added. On day 6, non-adherent and loosely adherent DC aggregates were harvested and sub-cultured overnight. On day 7, only cultures with >90% cells expressing CD11c by flow cytometry (FCM) were used.

### 4.4. Cell Viability Assay

The cytotoxicity assay of astaxanthin with different doses was performed in DCs using the CCK-8 kit in accordance with the manufacturer’s instructions. Briefly, 5 × 10^3^ cells were cultured in 96-well plate. After treatment, 10 μL CCK-8 was added to each well, and the cells were incubated for an additional 1 h. The absorbance was measured at 450 nm, and the results were compared as a percentage of the control group.

### 4.5. Cytokine Assay

In vitro, the DCs were incubated with astaxanthin and/or LPS for 24 h. Next, the levels of TNF-α, IL-6, and IL-10 in the culture supernatants were measured by using ELISA kits (eBioscience) and were performed according to the manufacturer’s instruction.

### 4.6. Phenotype Assay

DCs were harvested and washed twice with PBS, and incubated with FITC-MHCII, PE-CD40, PE-CD80, FITC-CD86, or their respective isotypes, at 4 °C for 30 min as per the manufacturer’s guidelines. After being washed three times with PBS, DCs were analyzed by FCM.

### 4.7. Endocytosis Assay

The harvested DCs were incubated with 1 mg/mL FITC-Dextran at 37 °C for 30 min as previously described [54]. After incubation, DCs were washed twice with PBS and analyzed by FCM. In addition, 4 °C control was also performed to exclude adhesion.

### 4.8. Migration Assay

The chemotaxis of DCs was performed in a 24-well transwell chamber (pore size, 5 μm; Corning) as described previously [55]. DCs (1 × 10^5^ cells) were then seeded onto the upper chambers and CCL19 (200 ng/mL) was added in the lower chamber. After incubation for 4 h, the migrated cells were collected from the lower chamber, and the number of cells was counted by FCM.

### 4.9. Allogeneic Mixed Lymphocyte Reaction Assay

Male BALB/c mice, 6 weeks old, were from the Animal Research Center of Yangzhou University (Jiangsu, China). Responder T cells were purified from mice splenic lymphocytes using a CD4^+^ T cell isolation kit and labeled with CFSE according to the manufacturer’s instructions. Next, these cells were cocultured in duplicate with DCs (DC/T cell ratios of 1:1 or 1:5) in 5% CO_2_ incubator at 37 °C for 5 days and detected by FCM.

### 4.10. HO-1 and Nrf2 Protein Expression Assay

The treated DCs were incubated with Alexa Fluor 647 HO-1, PE-Nrf2, or the respective isotypes for 30 min at 4 °C. The cells were analyzed using FCM.

### 4.11. Body Weight Change Assay

Six-week-old C57BL/6 mice were divided into five groups (*n* = 10/group). In the treatment group, the mice were given astaxanthin orally for 4 days every 24 h, and the doses of astaxanthin were 50, 100, and 200 mg/kg, respectively; 48 h after the firstly oral administration, the mice received LPS (10 mg/kg body weight) by intraperitoneal injection, body weight changes were monitored for 3 days.

### 4.12. Survival Rate and Cytokine Assay

48 h after 1st oral administration, the mice received LPS (20 mg/kg body weight) by intraperitoneal injection, survival rates were monitored for 40 h as described previously [56]. The mice were euthanized and blood was collected at 4 h after LPS injection, the levels of cytokines (TNF-α, IL-6, and IL-10) in plasma were measured by an ELISA kit according to the manufacturer’s protocol.

### 4.13. Statistical Analysis

Results were expressed as the means ± SD. Statistical significance between the 2 groups was determined by unpaired Student’s two-sided *t*-test. To compare multiple groups, one-way ANOVA with Tukey’s post hoc test was performed by using SPSS 17.0. * *p* < 0.05, ** *p* < 0.01.

## 5. Conclusions

In summary, our findings showed that astaxanthin inhibited the immune dysfunction of DCs induced by LPS via the activation of HO-1/Nrf2 axis in vitro, and enhanced the survival rate of LPS-challenged mice in vivo, which might be used as a potential candidate strategy for clinical sepsis.
